# Comprehensive review of SGLT2 inhibitors’ efficacy through their diuretic mode of action in diabetic patients

**DOI:** 10.3389/fendo.2023.1174692

**Published:** 2023-07-20

**Authors:** Cesar Ernesto Lam-Chung

**Affiliations:** Department of Endocrinology and Metabolism, Complejo Hospitalario Dr. Manuel Amador Guerrero, Colón, Panama

**Keywords:** SGLT2 inhibitors, diuretic action, diabetes, efficacy, canagliflozin, empagliflozin, dapagliflozin

## Abstract

SGLT2 inhibitors (SGLT2i) are now the mainstay therapy for both diabetes and heart failure. *Post-hoc* publications, meta-analysis, and conference presentations of the eight SGLT2i Cardiovascular Outcomes trials (CVOTS) done in diabetic patients constantly echo that this class of drug decreases mortality, reduces cardiovascular events, and prevents heart failure and kidney disease. This review of medical agencies’ SGLT2i analysis (FDA and EMA) helps to understand the reality of SGLT2i results in those trials, avoiding to consider observational and statistically undemonstrated endpoints as validated. They also confirmed the unique diuretic mode of action of SGLT2i, promoting osmotic diuresis, and its potential adverse events secondary to hypovolemia and hematocrit increase. They also support the understanding that the beliefs in SGLT2i morbi-mortality benefits are largely overstated mostly based on undemonstrated endpoints. Finally, it is clear that SGLT2i’s antidiabetic action, secondary to its renal mode of action, plateaued after a few months and decreased strongly over time, questioning its long-term goal of maintaining diabetic patients’ HbA1c below 7%. Also, this effect in patients with renal impairment is quasi null. We think that this review would be very helpful to every physician treating diabetic patients to better balance belief and reality of SGLT2i prescription effects.

## Introduction

SGLT2 inhibitors (SGLT2i), first identified as anti-diabetic drugs (1), are now recommended both by diabetes (2) and heart failure (3) guidelines, and a growing number of patients are exposed to them (4). *Post-hoc* (5, 6) publications, meta-analysis (7, 8), and congresses presentations (9–12) of the eight SGLT2i Cardiovascular Outcomes trials (CVOTS) done in diabetic patients (13–19) constantly echo that this class of drug decreases mortality, reduces cardiovascular (CV) events, and prevents heart failure and kidney diseases. Even the Expert Committee of the WHO explained the integration of empagliflozin in the Essential Medicine list «*based on high-quality evidence of reduced risk of all-cause mortality, major cardiovascular adverse events, and adverse renal outcomes, and a reasonable safety profile*» (20). However, explaining the mode of action of SGLT2i seems to remain complex, and many publications are estimating that SGLT2i’s mode of action is finally multifactorial (21–23), in that «*underlying mechanisms are not clearly understood*» (24) at the point that «*novel mechanisms have been proposed to explain these benefits*» (17, 18), resulting in a «*complex web of interacting effects starting in the kidney and modulating multiple metabolic pathways in a variety of other organs*» (25).

The United States Food and Drug Administration (FDA) and European Medicines Agency (EMA) reviews and analyses of medical drugs through submitted files from phase 1 to phase 3 trials are a neglected source of scientific evaluation of drugs’ mode of action and clinical trials, despite being open to the public. These official data from governmental organizations may help to draw a validated picture of SGLT2i’s mode of action and clinical efficacy in diabetic patients, which could interest prescribers.

## Method

Agency specialists’ reviews and analyses may help to shed light on the demonstrated mode of action and validated clinical endpoints of SGLT2i in studies and randomized clinical trials13-19. We reviewed all the US FDA and EMA SGLT2i files since first market authorizations to the last labeling extensions in diabetes. Such files are available on both agencies’ websites and include pharmaceutical, clinical, and safety reviews by specialists of each domain.

SGLT2i files presenting canagliflozin (26), dapagliflozin (27), empagliflozin (28), and ertugliflozin (29) were first submitted to the US FDA after 2010 to obtain an indication as anti-diabetic agent (year of obtention: Canagliflozin 2013, Dapagliflozin 2014, Empagliflozin 2014, Ertugliflozin 2017). Usual studies of diabetic patients, as well as of animals, were included to explain their mode of action, which were summarized by FDA reviewers. Other studies evaluated the different SGLT2i effects on HbA1c on short-duration studies, in monotherapy, and in combination with other anti-diabetics, a work completed by the preliminary examination of those drugs’ effects on CV events. Since March 2008, the FDA has also been requesting for post-marketing studies to demonstrate that a new therapy to treat type 2 diabetes is not associated with an unacceptable increase in CV risks. If classically, this solely suggests a non-inferiority versus placebo, SGLT2i safety trials also included superiority endpoints (13–17) and several phase 3 trials were also later achieved in diabetic patients with severe kidney disease (18, 19). Reviews of those trials are also available on the agencies’ websites.

## Results

### SGLT2i’s *mode of action as defined by agencies*

SGLT2i inhibits the sodium glucose cotransporter-2 (SGLT) receptor preventing glucose reabsorption in the proximal tubule of the kidney. This results in glucosuria and a decrease of serum hyperglycemia associated with diabetes. However, inhibition of the sodium glucose cotransporter-2 also provokes a natriuresis, or osmotic diuresis, decreasing blood pressure and increasing hematocrit. Adverse events may follow those two consequences of sodium glucose cotransporter-2 inhibition.

Dapagliflozin was described by the FDA in its 2014 Summary Review (30) as «both a diuretic and a glucose lowering agent that sets it apart from other anti-diabetic agents». A diuretic dose effect was identified, «the 5 mg dose produced less glycosuria and volume loss *than* the 10 mg dose». The volume loss was responsible for eGFR decrease and frequent adverse effects of hypovolemia and hypotension: «Modest dose dependent early (1 week) changes in serum creatinine and eGFR attributed to volume loss were *seen* in the clinical program. Hypovolemic/hypotensive events are directly related to the *drug’s* diuretic effect».

A study, belonging to the files submitted to the FDA (phase 2b MB102035) and published in 2013 (31), confirmed that dapagliflozin diuretic effect was more important than that of hydrochlorothiazide (HCTZ): «*Dapagliflozin 10 mg caused a greater decrease in (…) measured plasma volume compared to placebo and hydrochlorothiazide 25 mg at Week 12*». Dapagliflozin had a more potent effect on systolic blood pressure (SBP) than HCTZ (−5.6 vs. −4.9 mmHg), such as in the reduction in office systolic/diastolic BP (−12.3/−5.1 mmHg) compared to HCTZ (−7.2/−1.6 mmHg). Volume loss caused reductions in body weight in the first week, which plateaued in the HCTZ group at −1.1 kg, but decreased throughout follow-up in the dapagliflozin group till −2.4 kg at week 12. Decreased plasma volume with dapagliflozin led to a hematocrit increase of +2.2% in the dapagliflozin group, compared to −0.9% in the HCTZ group. The FDA stated that, «*taken together, these results imply that the reduction in BP with dapagliflozin therapy is associated with a ‘natriuretic/diuretic-like’ effect*», which was responsible for a significant loss in plasma volume. «*The results suggest a 7% reduction in plasma volume with dapagliflozin treatment indicating a diuretic effect possibly owing to enhanced sodium excretion or osmotic diuresis as a result of increased glucose excretion*». In consequence to this clear identification of SGLT2i diuretic effect linked to adverse events, the FDA reviewers proposed to use the lowest available dose for dapagliflozin initiation.

Similarly, canagliflozin’s FDA review (32) concluded that, «*as an osmotic diuretic, canagliflozin could also lead to adverse events related to reduced intravascular volume*». It was characterized that «*the incidence of volume depletion-related events with canagliflozin was dose-dependent and 2-3 fold higher compared to placebo»*. Dose-related risk of volume depletion adverse events was identified among various conditions, such as subjects with low baseline eGFR (<60 ml/min/1.73 m^2^), concomitant use of ACE inhibitors or ARB (with twofold increase of volume depletion events), concomitant use of diuretic, ≥65 years of age, >7.9% baseline HbA1c, ≤110 mmHg systolic blood pressure, diabetic complications, and ≥10 years’ duration of diabetes.

**Figure 1:**
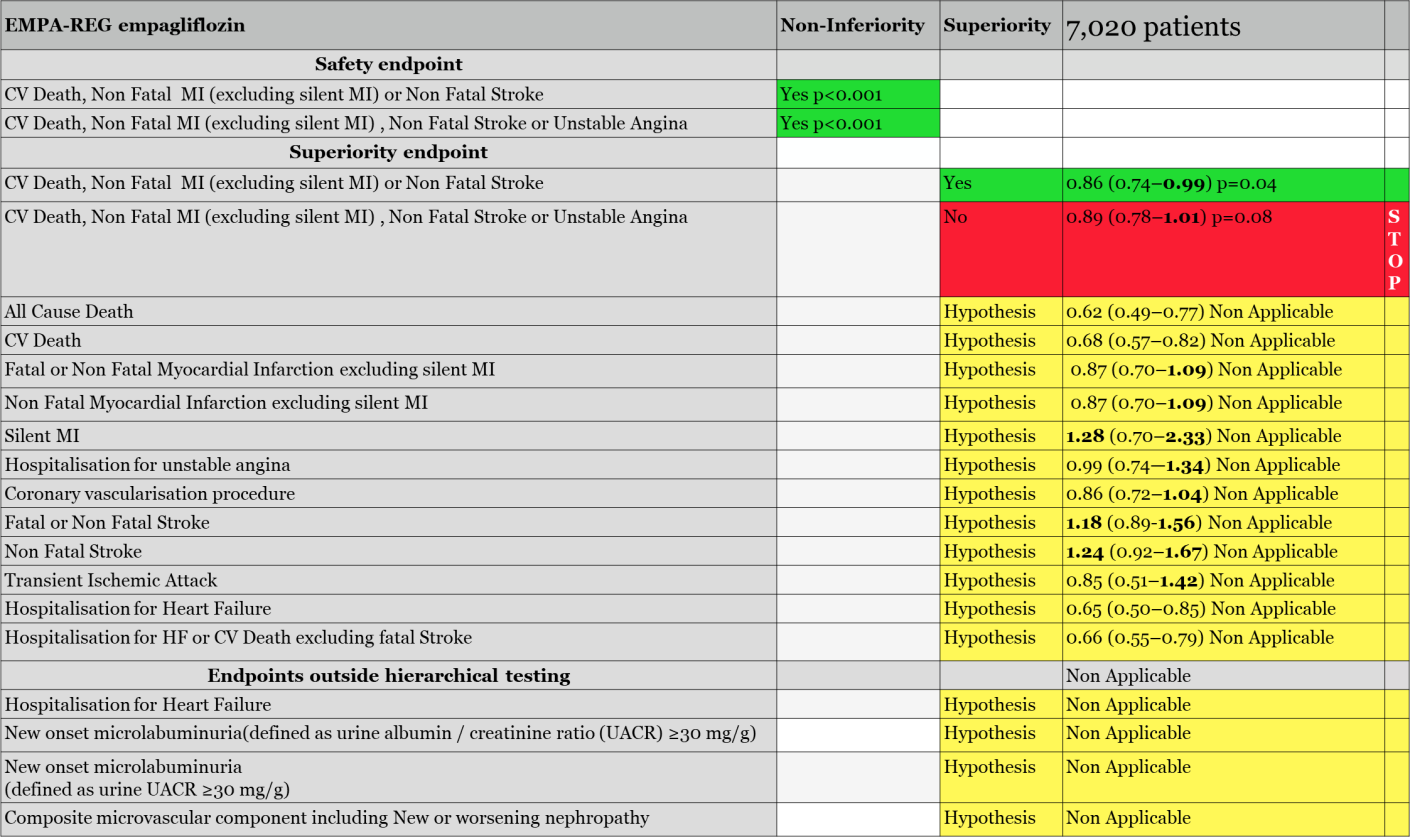
EMPA-REG hierarchical testing and results.

Same lines of evidence were drawn during empagliflozin review by the FDA: «Due to the diuretic effect of empagliflozin, the development of volume depletion is a theoretical concern» (33). Concomitant use of diuretic medications resulted in an increase in volume depletion and the incidence in the empagliflozin-treated patients was higher than *in* placebo-treated patients. When added to loop diuretics, there was a more noticeable increased incidence of these events with empagliflozin treatment compared to placebo. Empagliflozin’s significant increase in natriuresis versus placebo was later confirmed in a 2020 publication, particularly when combined with loop diuretics (bumetanide), resulting in a significant reduction in blood volume and plasma volume (34). Empagliflozin FDA reviewers also noted that «*Additional adverse events (AEs) seen more frequently with empagliflozin included dry mouth, thirst, and increased urination*»33. Increase in hematocrit was also seen in laboratory tests with a +2.8% increase (33). Interestingly, «*the increase in hematocrit with empagliflozin treatment appears reversible with discontinuation of treatment*» (33), linking it with volume depletion (**Figure 1**).

In a later FDA review published in 2018 (35), accumulation of data offered FDA reviewers a clearer view of the diuretic action of SGLT2is and related adverse effects:

«The *SGLT2* inhibitors, including empagliflozin, may be associated with osmotic *diuresis* and possible intravascular volume contraction, potentially predisposing patients to acute kidney *injury*, especially in individuals with impaired renal function, heart failure, elderly patients, or patients receiving loop diuretics, ACEIs, angiotensin receptor blockers (ARBs), and non-steroidal anti-inflammatory drugs (NSAIDs). A recent meta-analysis of 92 SGLT2 inhibitor clinical trials also reported an increased risk of volume depletion-related AEs (OR 1.20; 95% CI 1.10-1.31)» (36).

The potent SGLT2i diuretic effect occurring immediately after initiation also allowed to identify, in the development program studies, an early increased risk in stroke related to plasma volume decrease, hemoconcentration, and thrombosis. After a first identification during dapagliflozin review, FDA reviewers identified the same risk with canagliflozin: «*This is the second SGLT2 inhibitor program were an early imbalance in CV events is observed (…) these events could result from an acute effect related to drug initiation (i.e. volume contraction/hemoconcentration)» and «related to volume depletion is the potential for thrombosis, since canagliflozin had a osmotic diuretic effect which lead to hemoconcentration*». Among individual components*, «the point estimate for stroke was greater than 1 at 1.46, although the 95% CI was wide and crosses 1 (0.83, 2.58)* (3)*», also «most strokes with canagliflozin were ischemic in nature; 79% (37/47) and 56% (9/16) of strokes with canagliflozin and comparators were ischemic strokes respectively»* (3).

In conclusion, SGLT2i’s mode of action, as understood by US and European medical agencies, can be summarized: SGLT2i reduces reabsorption of glucose from the glomerular filtrate in the proximal renal tubule, along with a concomitant reduction in sodium reabsorption, leading to urinary excretion of glucose and osmotic diuresis. The natriuresis and osmotic diuresis provokes a reduction in plasma volume, which could lead to hypovolemia, increase in hematocrit, reduction in systolic and diastolic BP, and eventually weight reduction. Clinically, «*after taking SGLT2 inhibitors, the early manifestation is an increased urine volume in the first few days, then the urine volume returns gradually to a baseline level in several weeks, and a reduction of plasma volume of* ∼*7.3% is observed after 12 weeks* (1)*».* Interestingly, compared with loop diuretics, *«SGLT2 inhibitors tend to remove more fluid from the interstitial space than from the circulation, resulting in more electrolyte-free water clearance*» (37).

### SGLT2i’s effect on HbA1c in short-term studies

FDA’s dapagliflozin review of clinical studies in diabetic patients concluded that dapagliflozin provided superior glucose-lowering effect compared to placebo after 24 weeks in patients with normal or mildly impaired renal function: «*HbA1c reduction across trials and doses ranged from -0.40% to -0.84%*» when used as monotherapy or other anti-diabetic agents (30).

The HbA1c reductions with canagliflozin 100 mg and 300 mg relative to placebo were −0.91 and −1.16% as monotherapy, and −0.62 to −0.92 as add-on to other anti-diabetic agents. Notably, increased dose of canagliflozin did not provide an additional glycemic effect. «*The additional incremental HbA1c lowering with 300 mg relative to 100 mg was about 0.1 to 0.15% with a couple of trials showing difference of 0.2%, and this additional HbA1c lowering may not be beneficial in the context of increased safety issues with 300 mg dose*» (32).

Empagliflozin was also shown to be effective in reducing HbA1c as monotherapy and as add-on to other anti-diabetic regimens with mean placebo-adjusted change ranging from −0.48% to −0.73% with the 10-mg dose, and from −0.59% to −0.84% with the 25-mg dose. Moreover, «*while the 25 mg dose demonstrated a numerically greater reduction in HbA1c than the 10 mg dose, the difference between the two doses is of questionable statistical and clinical significance*» (33).

Finally, SGLT2i’s renal mode of action also explained why diabetic patients with impaired renal function modestly benefit SGLT2i in terms of glycemia control: «*Attenuation of glycemic efficacy with canagliflozin in patients with renal impairment is expected since urinary glucose excretion by canagliflozin depends on the renal threshold for glucose, plasma glucose level, and renal function. With diminished renal function, canagliflozin’s effect on urinary glucose excretion would be expected to be reduced*» (32). A modest efficacy was effectively observed in such patients with placebo-subtracted mean reductions of −0.3% and −0.4% with the 100-mg and 300-mg doses, respectively. Similarly, «*dapagliflozin was not observed to have statistically or clinically important glucose lowering effect in patients with moderate renal impairment*» with an HbA1c reduction of −0.11% (*p* = 0.435) at 10 mg and −0.08% (*p* = 0.51) at 5 mg1.

### FDA/EMA post-marketing safety trials reviews

Safety trials are mandatory for drugs getting an approval for diabetes treatment from the FDA since 2008. The objective is to demonstrate non-inferiority versus placebo on main CV outcomes, CV death, non-fatal MI, and non-fatal stroke (MACE), with a non-inferiority margin of 1.3. The first six SGLT2i trials, EMPA-REG (5), CANVAS and CANVAS-R (6), DECLARE-TIMI7, VERTIS (8), and SCORED (17), were thus designed as safety trials. All these trials followed a statistical method called hierarchical testing (38). Shortly, a list of endpoints is ranked in a hierarchical order. The statistical power exists to analyze the first endpoint. If the first endpoint is significantly improved, analysis can go on to the next endpoint in the hierarchy with sufficient statistical power. However, as soon as an endpoint is not improved significantly, the hierarchical testing stops and all remaining endpoints are only considered as hypothesis (observational endpoint), as there is no more sufficient statistical power to pursue. It explains why, if the effect on an endpoint placed after the *stop* is evaluated and rated a *p* < 0.05, this endpoint must be considered only as a hypothesis. This hierarchical testing strategy is highly important to understand what was really validated and what remained a hypothesis, thus needing a new trial to confirm the findings of the safety SGLT2i trials.

### EMPA-REG trial evaluating empagliflozin

The EMPA-REG trial, evaluating empagliflozin, was the first finalized SGLT2i safety trial. Notably, it included 99% of diabetics with confirmed CV disease, and 8% with diagnosed heart failure. However, heart failure was not evaluated at baseline, and heart failure therapies were neither requested to be consistent with best practice. As reported by the FDA (39) and EMA (40), during the trial, the primary endpoint was changed. If the primary endpoint of the trial was the classical MACE, CV death, non-fatal MI, and non-fatal stroke, it was modified to exclude «*silent MI*» from the non-fatal MI endpoint definition. The primary endpoint became CV death, non-fatal MI (excluding silent MI) or non-fatal stroke. A secondary primary endpoint called MACE-plus, adding to the previous one, unstable angina, was next in the hierarchical testing. Those two endpoints had first to be validated in terms of non-inferiority versus placebo before being tested for superiority versus placebo if validated.

The primary and the secondary safety endpoints of EMPA-REG were significantly reduced for non-inferiority to placebo (*p* < 0.001) allowing us to evaluate them for superiority. The MACE (excluding silent MI) was found to be statistically superior to placebo with an upper limit of 0.99, 0.86 (0.74–0.99), *p* = 0.04. However, the secondary primary endpoint for efficacy, CV death, non-fatal MI (excluding silent MI), non-fatal stroke, or unstable angina, was not significant and closed the hierarchical testing: «*Had there been any remaining hypotheses to be tested in the hierarchy they would principally be considered as exploratory or hypothesis generating»* commented the FDA reviewers of the EMPA-REG trial (**Figure 1**), making it clear that all the remaining endpoints, placed after the *stop* of the hierarchical testing, or placed outside of the hierarchical testing, such as all-cause death, CV death, hospitalization for heart failure, hospitalization for heart failure, or CV death excluding fatal stroke, and every renal endpoints remained *hypothesis* only, needing a new trial for validation.

Regarding the hospitalization for heart failure endpoint, FDA reviewers commented that, «as previously noted, hospitalization for HF and other HF-related endpoints were *not* included in a plan to control the overall *Type* 1 error; hence, all of these analyses are

exploratory», explaining that, «because of its diuretic effect, it is certainly plausible that empagliflozin could reduce the risk of HF hospitalization (in patients with a preserved or reduced EF); however, we believe this hypothesis should be confirmed in a well-designed and well conducted trial in patients with HF».

Regarding renal endpoints, FDA reviewers eliminated also any positive conclusion from EMPA-REG results: «There were too few clinical events to draw meaningful conclusions that differences between therapies truly existed», adding that «multiple changes to *the* renal endpoints definitions in the protocol and used for the purpose of *exploratory analyses* occurred over the course of the trial»; moreover «renal endpoints were *not adjudicated*, and also were *not prespecified*. There was no control for type 1 error»_40_.

EMPA-REG agencies’ reviews concluded that only one efficacy endpoint, CV death, non-fatal MI (excluding silent MI), or non-fatal stroke, may be considered as significant. It was only driven by CV death reduction without any beneficial effect on myocardial infarction or stroke. Result by dosages of this endpoint, which was initially planned, was also replaced by a combined analysis during the course of the trial. Both were not significant, with *p* = 0.07 for empagliflozin 10 mg and *p* = 0.08 for 25 mg (39).

The FDA also noted that 40% of the CV death events were in fact not CV deaths, but «*not assessable* deaths», adjudicated neither to CV death nor to non CV death. By protocol, they were added to CV death: «*More than one-third (40.1%) of all CV deaths are labeled as ‘fatal event not assessable’ which was defined as all deaths not attributed to the specified categories above and not attributed to a non-cardiovascular cause. It is not clear whether these events are truly CV deaths*», adding that, if this is common practice in CV studies, well-managed trials have less non-assessable deaths. Reviewers reevaluated the primary endpoint without those non-assessable deaths: «*In a sensitivity analysis that removes all “non-assessable” deaths from the primary endpoint, empagliflozin was no longer demonstrated to be superior to placebo (HR 0.90, 95% CI 0.77, 1.06)»*.

Additionally, FDA reviewers questioned the late exclusion of silent MI from the MACE endpoint, as silent MI is common in diabetic patients. In EMPA-REG, the silent MI endpoint was not in favor of empagliflozin: «*53 experienced a silent MI, 15 (1.2%) in placebo and 38 (1.6%) in empagliflozin»* with HR 1.28 (0.7, 2.33). In a sensitivity analysis, FDA reviewers evaluate again the primary endpoint, not excluding the silent MI events, and again, the primary endpoint became non-significant, HR 0.91 (0.73, 1.13). The EMA added, «*The fact that the primary endpoint was reworded regarding silent MIs raised questions as to whether this could have been a modification potentially based on unblinded data, and not a clarification as described in the study report*» (40).

The non-fatal stroke endpoint showed an increase on average by 24% in comparison with placebo, HR 1.24 (0.92, 1.67), and fatal and non-fatal stroke by 18%, HR 1.18 (0.89, 1.56), with more events in the empagliflozin group. If both did not reach significance on the global population, the FDA reviewers highlighted «*sub-groups where the risk reached nominal statistical significance*», subjects <65 years of age, HR 1.6 (1.03, 2.49); subjects with a baseline HbA1c ≥8.5%, HR 2.13 (1.21, 3.74); subjects treated with insulin, HR 1.57 (1.03, 2.41); and European population, HR, 2.06 (1.26, 3.29). European population represented more than 40% of the study population. EMA published the Kaplan–Meier curve of stroke in the European population, which established this doubling risk of stroke with empagliflozin in European diabetic patients and hypothesized that «*the excess of strokes during treatment with empagliflozin (if not chance) may be partly related to the decrease of circulating blood volume, which can be seen as an increased hematocrit in the empagliflozin treated groups*», linking the increase in stroke risk to the rapid osmotic diuresis, reducing blood volume and increasing hematocrit. Notably, the primary endpoint of EMPA-REG was not reduced in the European population, 1.02 (0.81, 1.28), due to this increase in stroke and the absence of effect on CV death.

None of the agencies accepted to validate the significance of the primary efficacy endpoint due to the absence of beneficial effect on myocardial infarction and stroke. However, the FDA finally accepted to consider the CV death endpoint as it was a component of the primary MACE endpoint, and also the component driving the endpoint. Looking at the type of CV death, heart failure death was the only one significantly different in empagliflozin-treated patients vs. placebo (**Figure 1**). Note also that EMPA-REG primary endpoint was not significant in the North American population.

In Europe, the EMA did not grant empagliflozin additional market authorization and explained its decision: «in the case of the current empagliflozin application based on a single pivotal trial, some further considerations were:

1. *EMPA-REG* was primarily a safety study and the primary endpoint resulted in a p *for* superiority of *only* 0.04,
2. Patients with established cardiovascular disease are only a subgroup of the total (T2DM) population with overlap between the two indications claimed,
3. The effect on the MACE-3 endpoint was *inconsistent*, with an increase in stroke» (40).

Both agencies also concluded that the effect seen was independent from HbA1c reduction. HbA1c difference generated by empagliflozin versus placebo after 3.1 years was −0.24% with empagliflozin 10 mg and −0.36% with 25 mg.

In conclusion, the EMPA-REG trial demonstrated that empagliflozin was non-inferior to placebo on MACE risk and was superior to placebo regarding CV death. Any other endpoint remained a hypothesis.

### *CANVAS/CANVAS-R (*the *CANVAS program) evaluating canagliflozin^42^*

The CANVAS program evaluating canagliflozin in diabetic patients included 65% of patients with CV disease; 15% had heart failure. The CANVAS program was a combination of two studies, the CANVAS trial initiated in 2009 (4,330 patients) and the CANVAS-R trial initiated in 2014 (5,812 patients). CANVAS-R was first planned to study the effect of canagliflozin on kidneys with a primary endpoint on albuminuria. However, the plan changed when CANVAS data were unblinded secondary to the identification of a +4.5% to +8.5% increase in LDL-cholesterol. Additionally, in a safety analysis including CANVAS data, an early increase imbalance in stroke not favoring canagliflozin was found, with HR 1.46 (0.83, 2.58). Most of these strokes were ischemic. FDA reviewers commented that «*the early events might be attributed to a high-risk population being more sensitive to drug-induced volume changes*».

Because of the uncertainty that CANVAS was still appropriate to demonstrate safety of canagliflozin versus placebo, a second study, CANVAS-R, replicating the CANVAS protocol, was initiated, whose results would be combined to CANVAS in a meta-analysis.

On 17 March 2016, the FDA received a safety report of CANVAS related to a twofold increase in amputation risk with canagliflozin. Trial was not stopped and subsequent updates on 17 March 2017 and 10 April 2017 continued to show an increase in the risk of lower limb amputations in CANVAS, with a similar increased risk in CANVAS-R. On 25 July 2017, the labeling for canagliflozin (as other SGLT2i) was updated to add a Boxed Warning to warn healthcare professionals about the risk of lower limb amputation.

**Figure 2:**
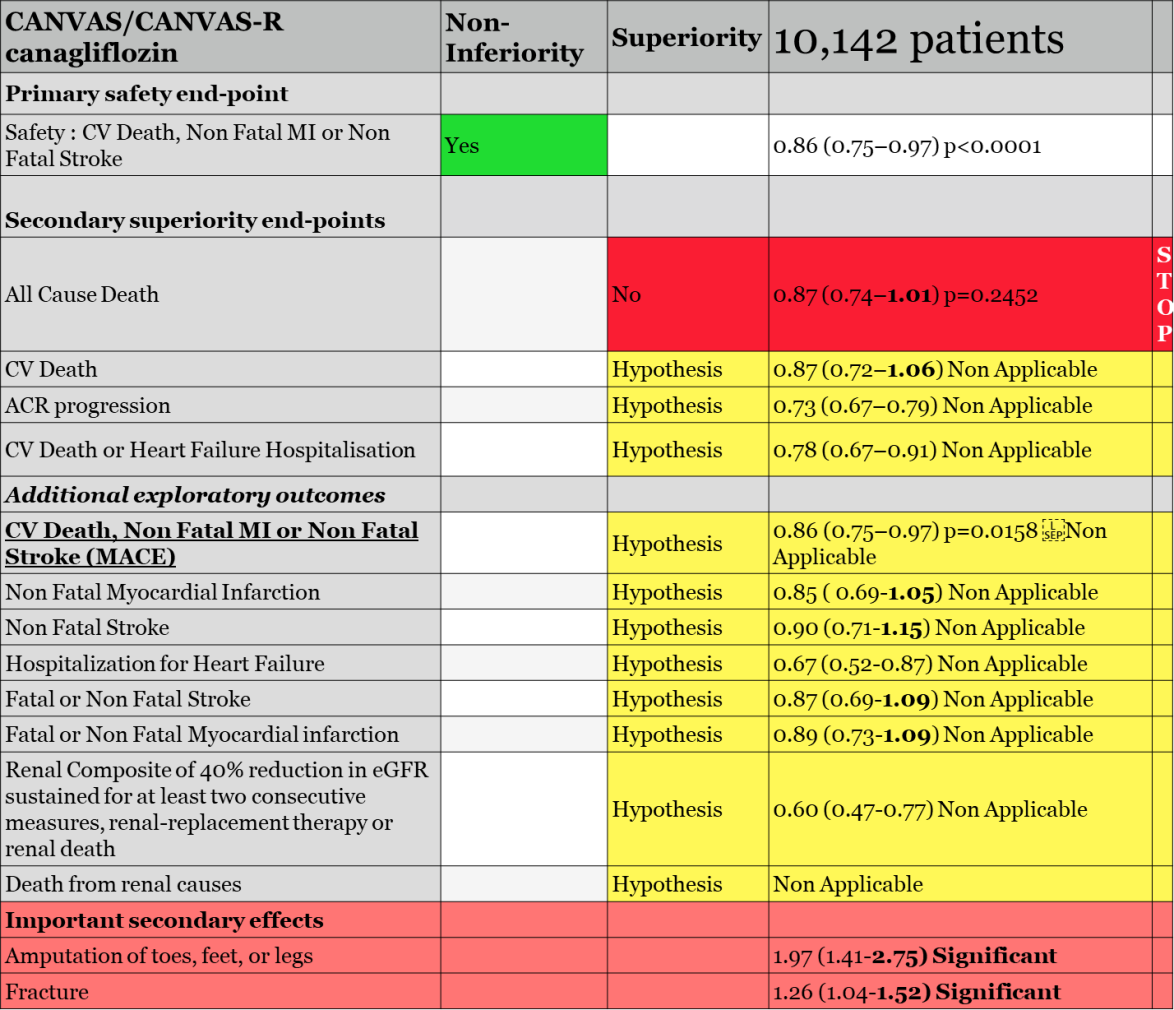
CANVAS/CANVAS-R hierarchical testing and results.

For CANVAS/CANVAS-R meta-analysis, the chosen sequence of hierarchical testing was first, non-inferiority for MACE (CV death, non-fatal MI or non-fatal stroke) with a 1.3 margin, followed by superiority testing of canagliflozin for all-cause mortality, then superiority testing for CV death. Reviewers noted that «*the rationale for the hypothesis tests apparently had to do with the sponsor knowing the results of the EMPA-REG OUTCOMES study which demonstrated a reduction in CV death vs. placebo*» (**Figure 2**).

Statistical analysis conducted by FDA reviewers confirmed the validity of the first MACE endpoint for non-inferiority of canagliflozin versus placebo, allowing us to test the first endpoint for superiority according to hierarchical testing, all-cause death. All-cause death, with 281/4.347 (6.5%) for placebo and 402/5.795 (6.9%) for canagliflozin, was not significant, HR 0.87 (0.75, 1.02), *p* = 0.24. The statistician of the FDA confirmed that, «S*uperiority on all-cause mortality was the second hypothesis to be tested in the hierarchy. Since the testing scheme was sequential, all of alpha is spent and subsequent endpoints were not tested*», meaning that all remaining endpoints could only be considered as hypotheses. They evaluated the CV death endpoint anyway, *«although further hypothesis testing was stopped, canagliflozin did not reduce the risk of cardiovascular death compared to placebo; the hazard ratio was 0.96 (95% CI: 0.77, 1.18)*». FDA statisticians also reviewed the causes of death in the trial. CV death accounted for 64% of total death and «*among CV deaths, incidence rate of death due to acute myocardial infarction (…) appear to be almost twice higher with canagliflozin compared to placebo (2.34 vs 1.25/1000 PY)*». Moreover, rate for fatal/non-fatal MI and fatal/non-fatal stroke were not significantly increased. The incidence rate of heart failure death or cardiogenic shock was also higher in canagliflozin compared to placebo (2.94 vs. 2.43/1000 PY).

Regarding the heart failure endpoint, time to first occurrence of hospitalization for heart failure, a hypothesis, was «*nominally significant*», HR 0.67 (0.52, 0.87), and «*subgroup analysis showed that the reduced risk of HR for time to the first occurrence of hospitalization for heart failure for canagliflozin was mainly seen in those with history of heart failure at baseline*». FDA reviewers commented that «*a decrease in heart failure events is plausible given the mechanism of action for canagliflozin, which has a diuretic effect. However, it should be noted that CANVAS and CANVAS-R enrolled a broad population of heart failure patients, and patients with heart failure were not directed to optimize their medical therapy for heart failure before enrolment and during the study. As discussed before, heart failure was an exploratory endpoint in these studies*».

Regarding HbA1c, the mean baseline HbA1c was similar between treatment groups, 8.24% in the placebo and 8.25% in the canagliflozin group. Despite an initial reduction, after week 52, HbA1c levels in the canagliflozin group appeared to rise slowly, and at week 104, the difference between groups was −0.47%. Also, the decline in body weight was noticeable up to week 26, after which there was a progressive smaller decline, up to the end of the trial, in the canagliflozin treatment group.

Despite the negativity of the meta-analysis hierarchical plan, the sponsor requested to the FDA a new indication for canagliflozin regarding MACE reduction, CV death, non-fatal MI, or non-fatal stroke, an exploratory but not demonstrated endpoint according to the protocol.

Note that this MACE endpoint in CANVAS and CANVAS-R trials, taken separately, was not significant, HR 0.88 (0.75, 1.03) and HR 0.82 (0.66, 1.01), respectively. Also, none of the three endpoints taken separately, CV death, non-fatal MI, or non-fatal stroke, were reduced in any of the two trials.

Evaluation of time to first adjudicated MACE of canagliflozin vs. placebo with meta-analysis data showed a nominal significant reduction, HR 0.86 (0.75, 0.97), *p* = 0.0158. However, none of the individual components showed statistical significance. Additional analysis by dosage of canagliflozin 100 mg and canagliflozin 300 mg also showed non-significant results, HR 0.85 (0.72, 1.0) and HR 0.86 (0.66,1.13), respectively.

Although not pre-specified in the Statistical Analysis Plan, FDA would accept the sponsor proposal as «this evidence was considered to support the claim that canagliflozin is superior to placebo in reducing the overall risk for MACE». However, the FDA statistician reviewer testified that «*because the superiority of canagliflozin compared to placebo in MACE was not pre-specified in the meta-analysis, and because the superiority met statistical significance based on meta-analysis of two CV studies and not seen in each individual study, I had some concerns whether the results of this meta-analysis were statistically robust and* considered to be sufficient evidence of CV benefit, as this meta-analysis would be considered to be ‘one study’ and we typically require more than one adequate and well-controlled study to support a claim for a new benefit/indication».

On 29 October 2018, the FDA approved canagliflozin with a new indication «*to reduce the risk of major adverse cardiovascular events in adults with type 2 diabetes mellitus who have established cardiovascular disease*». To be noted, the FDA decided anyway not to cite the MACE endpoints. EMA did not change the labeling for canagliflozin indication.

In conclusion, CANVAS/CANVAS-R demonstrated non-inferiority of canagliflozin versus placebo with an upper limit of 1.3. Statistically, the MACE endpoint being not prespecified and not validated by hierarchical testing cannot be considered as significant and remains a hypothesis with no meaningful clinical benefit.

### DECLARE-TIMI evaluating dapagliflozin

The DECLARE-TIMI trial recruited 17,190 diabetic patients followed up for 5 years; 41% had established CV diseases. For safety evaluation, the primary endpoint was the classical MACE. Following EMPA-REG results, a protocol amendment included two co-primary efficacy outcomes: first, CV death or hospitalization for heart failure, and second, the MACE. Secondary efficacy endpoints were, first, a composite renal endpoint, and second, all-cause death.

The primary safety MACE endpoint with HR 0.63 (0.84, 1.03) confirmed the non-inferiority of dapagliflozin versus placebo (*p* < 0.001), allowing us to test for superiority in the two primary efficacy endpoints, CV death or hospitalization for heart failure, and the MACE endpoint. CV death or hospitalization for heart failure was significantly reduced but «*driven entirely by the difference in events of hospitalization for heart failure (HR 0.73; 95% CI 0.61, 0.88)*». There were 212 (2.5%) patients in the dapagliflozin group and 286 (3.3%) patients in the placebo group in 5.1 years of the trial adjudicated for hospitalization for heart failure.

According to FDA reviewers, «it is mechanistically plausible that dapagliflozin could reduce the risk for hospitalization for heart failure given its diuretic effect», highlighting anyway that the trial did not collect baseline heart failure data, with no plan to optimize heart failure medications, and that NYHA IV patients were excluded from the trial. FDA reviewers also noticed that if «Lower incidence of HHF was observed (…) by subgroup based on history of *HF. However*, it is difficult to interpret the subgroup results based on HF history or baseline left ventricular ejection fraction because baseline HF status was not *well-*characterized at the start of the trial, and baseline drug/device treatment was not specified to be optimized prior to randomization (…). Some patients who experienced HHF events during the *trial* probably had undiagnosed heart failure at baseline, or baseline HF information was not adequately captured during the enrolment process. Therefore, subgroup analyses based on HF history do not provide information that would inform labelling of dapagliflozin for *a* HHF indication in any subpopulation of the DECLARE trial».

CV death was similar in the two groups. FDA reviewers commented that CV death endpoint in DECLARE-TIMI did not include non*-*assessable Death: «With regard to causes of death that were “undetermined” in this trial, 13.8% (73/529) and 14.6% (83/569) deaths in the dapagliflozin and placebo arms, respectively, fell into this category. This proportion of undetermined deaths was similar to that observed in the CANVAS program; *a* significantly higher proportion of deaths were considered “not assessable” in the *EMPA-*REG OUTCOME trial (approximately 40%). Unlike the *EMPA-REG* OUTCOME trial *and* CANVAS program, the DECLARE trial did not consider “undetermined” deaths as CV deaths for the purpose of calculating the primary e*ffi*cacy variables».

The next endpoint, the MACE, was not significantly reduced (*p* = 0.172), closing the hierarchical testing: «*superiority of dapagliflozin to placebo for MACE was not demonstrated; therefore, testing stopped at this point and secondary endpoints were not formally evaluated for statistical significance*». The renal composite endpoint was then considered exploratory due to the stop of the hierarchical testing with insufficient preserved alpha to allow for formal testing, as all-cause mortality, which anyway with HR 0.93 (0.82, 1.04), was not nominally significantly reduced.

Regarding HbA1c efficacy, the largest difference in treatment arms, −0.7%, was observed at 6 months post-randomization. At 4 years, the difference between arms was −0.24% (comparable to the difference observed in the EMPA-REG trial at week 206 (41), and the proportion of patients with HbA1c <7% was similar between dapagliflozin and placebo arms (28.7% vs. 26.6%).

In conclusion, dapagliflozin in DECLARE-TIMI fulfilled the requirement to not increase MACE endpoint in diabetic patients demonstrating non-inferiority versus placebo. Additionally, dapagliflozin demonstrated a treatment effect on reducing the risk for hospitalization for heart failure but not CV death. Note that no renal effect was attributed to dapagliflozin following DECLARE-TIMI analysis. FDA added the indication «*Reduce the risk of hospitalization for heart failure in adults with type 2 diabetes mellitus and established cardiovascular disease or multiple cardiovascular risk factors*» to dapagliflozin labeling. EMA did not, but SmPC was amended to include information on the outcome of the DECLARE trial.

### VERTIS evaluating ertugliflozin

The VERTIS trial included 8,246 diabetic patients, 100% with established CV diseases and 23.7% with heart failure, a population sample very similar to EMPA-REG. On average, the duration of the trial was 3.5 years. The primary endpoint included in the hierarchical testing was the MACE endpoint. With HR 0.97 (0.85, 1.11), ertugliflozin demonstrated to be non-inferior to placebo for safety but not for superiority, stopping the hierarchical testing; all remaining endpoints were considered hypotheses only.

No individual endpoint of the MACE was significantly reduced, with HR 0.92 (0.77, 1.11), *p* = 0.39 for CV death; HR 1.0 (0.86, 1.27), *p* = 0.66 for non-fatal MI; and HR 1.0 (0.76, 1.32), *p* = 0.99 for non-fatal stroke.

The heart failure hospitalization endpoint occurred in 2.5% and 3.6% in the treated and the placebo populations, respectively, HR 0.70 (0.54, 0.90), but remained a hypothesis. The endpoint, renal death, dialysis/transplant, or doubling of serum creatinine, which remained also a hypothesis, was not significantly reduced, HR 0.81 (0.63, 1.04), *p* = 0.08.

### SCORED evaluating sotagliflozin

The SCORED trial included 10,584 diabetic patients, 50% with a history of CV disease and 20% with heart failure; mean eGFR was 44 ml/min/m2. The trial ended earlier than planned as the sponsor decided to stop its participation. Approximately 30% of events were not adjudicated, which profoundly undermined the validity of clinical effect.

The primary endpoint, CV death, hospitalization for heart failure, or urgent visits for heart failure, was found significant for non-inferiority versus placebo and for superiority, HR, 0.74 (0.63, 0.88), *p* < 0.001. There was no significant effect on CV death, and the endpoint was mainly driven by a heart failure effect. The non-significant effect of CV death closed the hierarchical testing. Total death was exploratory and nevertheless nominally non-significant, HR 0.99 (0.83, 1.18).

## Discussion

After six randomized double-blind trials involving 57,000 diabetic patients, hierarchical testing strategy identified a superiority on CV death reduction effect with empagliflozin in the MACE endpoint, no superiority effect of canagliflozin, a reduction of hospitalization for heart failure superiority effect with dapagliflozin, no beneficial effect with ertugliflozin, and a hospitalization for heart failure reduction effect with sotagliflozin, but questionable as it is without full adjudication. All other endpoints either have been found without significance versus placebo or were not applicable as they were placed after the stop of the hierarchical testing or even placed outside of the hierarchical testing itself. All those endpoints remain not validated and are, thus, hypotheses only. The three benefits have been identified as independent from HbA1c. These results have been clearly highlighted by FDA and EMA reviewers.

FDA offered three market authorizations, one on CV death reduction for empagliflozin, one in cardiac event reduction for canagliflozin, and one in heart failure reduction for dapagliflozin.

All these three endpoints are fully compatible with the previously identified diuretic mode of action of SGLT2i and have obviously, as recognized by authors and agencies, nothing to do with the modest glycemic effect versus placebo recorded in all SGLT2i safety trials. Regarding the two results recorded for reduction of heart failure hospitalization, a diuretic effect has the potential to explain it, as it was summarized by agency reviewers.

Looking at the global results picture, what did the diuretic mode of action of empagliflozin teach us in EMPA-REG? Patients on empagliflozin benefited from a little advantage on BP. reduction (135.3 mmHg to 131.3 mmHg) but received no benefit in stroke or MI reduction. In contrast, they obtained a small increase in LDL and higher silent MI events (5). They lost 2–3 kg of weight, probably only water, a hypothesis consistent with its diuretic mode of action, and with the loss in weight highlighted by studies submitted to agencies. They benefited from a little reduction in HbA1c but without being controlled <7%, and progressively returned nearly to their baseline level of HbA1c. Potentially, always related to its mode of action, empagliflozin somehow benefited patients with heart failure, diagnosed and undiagnosed. If approximately 8.8% of the population had diagnosed heart failure, there was no screening for it. Knowing that among diabetic patients, prevalence of heart failure is usually 20% to 30% (42), it is possible that many heart failure patients were ignored, more particularly in Asia and South America, regions with higher Gini coefficients where patients often received less benefit from baseline heart failure guideline-recommended therapies, and suffered from a significantly higher risk of CV death or hospitalization for heart failure (43, 44). These two regions were also the only regions where EMPA-REG primary endpoint was found significant.

In a *post-hoc* analysis, it was shown that the difference between empagliflozin and placebo became numerically nominally significant after only 17 days for hospitalization for heart failure, and after 59 days for CV death (45). This result may be compatible with a diuretic effect in patients with untreated or inadequately treated heart failure. Another *post-hoc* analysis showed that patients with heart failure were the first beneficiary: after only 12 weeks, between empagliflozin and placebo in the heart failure and non-heart failure population, there was already a difference in hospitalization for heart failure [0 (0%) vs. 7 (2.9%) and 5 (0.1%) vs. 3 (0.1%)] and HHF or CV death [1 (0.2%) vs. 10 (4.1%) and 9 (0.2%) vs. 8 (0.4%)], an effect compatible only with a diuretic mode of action (46). In addition, the only significant difference among the different causes of CV death was heart failure death, which occurs in patients with heart failure. In total, the CV death reduction of EMPA-REG may potentially be chance finding. Effectively, the trial included heart failure patients, badly treated or not treated because they are undiagnosed. Also, especially for heart failure, an important difference in terms of survival has been demonstrated in trials between Europe/US and South American/South Asian countries, and the CV death endpoint was not significant in Europe and North American populations. Finally, SGLT2i mainly works with a diuretic mode of action, while their antidiabetic effect was judged as having no influence on results. Chance is not easily replicable.

Also, in the same time period, there was already an increase in stroke in the European population, a phenomenon also compatible with the empagliflozin diuretic mode of action as hypothesized by the FDA/EMA reviewers secondary to hemoconcentration, hematocrit increase, and thrombosis. Why is there a difference between regions? In Europe, as previously highlighted, heart failure may have been better detected and/or better treated. Adding a diuretic action able to increase hematocrit may have harmed the better-treated heart failure European patients but may have been beneficial to less well-diagnosed and treated ones in developing countries with a higher Gini coefficient, where out-of-pocket medicine is frequent. If we have the knowledge that diuretics do not reduce CV death in heart failure patients, this knowledge has been acquired in patients who have been recommended HF therapy, but what happens if a diuretic is all what you have been prescribed? In EMPA-REG, there was no baseline treatment control for heart failure, which may have allowed a diuretic to benefit patients and delay complications. In an exploratory analysis from the EMPA-REG trial, changes in markers of plasma volume were the most important mediators of the reduction in risk of CV death with empagliflozin versus placebo: Changes in hematocrit and hemoglobin, secondary to plasma contraction, mediated 51.8% and 48.9%, respectively, of the effect of empagliflozin versus placebo on the risk of CV death on the basis of changes from baseline, with similar results in analyses on the basis of updated means (47). Smaller mediation effects (maximum 29.3%) were observed for uric acid, fasting plasma glucose, and HbA1c, strongly emphasizing the importance of the empagliflozin diuretic effect and reduction in plasma volume on the CV death endpoint effect, also explaining why no beneficial effect was found on myocardial infarction and stroke, which may have been armed by it.

The absence of replicability of the CV death reduction endpoint in EMPA-REG, either by empagliflozin in later trials or by other SGLT2i, may plead for a chance effect. If not, this beneficial effect would have been replicated. In the VERTIS trial, evaluating ertugliflozin, another SGLT2i, in the same 100% CV risk population as EMPA-REG, there were no reduction in CV death in any endpoint, but again, a hypothesis on heart failure hospitalization needs to be confirmed. Exactly what would EMPA-REG have produced without the exclusion of silent MI? In fact, this benefit of EMPA-REG on CV death will never be replicated in any diabetic patient trial by any SGLT2i. Also, empagliflozin will fail to reproduce it in its own trials, particularly in EMPEROR-reduced in heart failure patients (48).

## Conclusion

Whatever the hypothesis, SGLT2i have been clearly identified as a diuretic by both the FDA and EMA. Looking at EMPA-REG clinical and biological results, an additional mode of action does not seem to be needed. Acting on kidneys through osmotic diuresis, empagliflozin favors glucose extraction, reducing glycemia and HbA1c, but only on the short term, as this whole effect rapidly plateaued and then diminished, making SGLT2i a «modest» anti-diabetic agent, as defined by the EMA.

If significance of the EMPA-REG efficacy primary endpoint was a surprise, we saw that it became secondary to several changes, including exclusion of silent MI and inclusion of additional 40% of non-assessable deaths, which of course would strongly compromise its replication in succeeding trials. No other cardiac or vascular endpoint, including myocardial infarction and stroke, was significantly reduced. Any renal or heart failure claims could not be considered and need to be demonstrated in other trials.

From now on, when reading an SGLT2i publication stating, for example, that «In the *EMPA-* REG OUTCOME trial, in patients with type 2 diabetes with established atherosclerotic cardiovascular disease, empagliflozin reduced the risk of hospitalization for HF by 35% (HR [95%CI] 0.65 [0.50-0.85]), CV death/HHF by 34% (0.66 [0.55-0.79]), and CV death *by*

*38% (0.62 [0.49-0.77])» (* 17), physicians would know what has been statistically demonstrated and is clinically relevant, and what is just a hypothesis awaiting confirmation.

## Author contributions

The author confirms being the sole contributor of this work and has approved it for publication.
